# Environmental Factors in an Ontario Community with Disparities in Colorectal Cancer Incidence

**DOI:** 10.5539/gjhs.v6n3p175

**Published:** 2014-03-24

**Authors:** Jeavana Sritharan, Rishikesan Kamaleswaran, Ken McFarlan, Manon Lemonde, Clemon George, Otto Sanchez

**Affiliations:** 1 Faculty of Health Sciences, University of Ontario Institute of Technology, Oshawa, Ontario, Canada; 2 Faculty of Business and Information Technology, University of Ontario Institute of Technology, Oshawa, Ontario, Canada; 3 Lakeridge Health, Oshawa, Ontario, Canada

**Keywords:** colorectal cancer, cancer disparities, community based research, environmental health, tobacco smoking, alcohol drinking

## Abstract

**Objective:**

In Ontario, there are significant geographical disparities in colorectal cancer incidence. In particular, the northern region of Timiskaming has the highest incidence of colorectal cancer in Ontario while the southern region of Peel displays the lowest. We aimed to identify non-nutritional modifiable environmental factors in Timiskaming that may be associated with its diverging colorectal cancer incidence rates when compared to Peel.

**Methods:**

We performed a systematic review to identify established and proposed environmental factors associated with colorectal cancer incidence, created an assessment questionnaire tool regarding these environmental exposures, and applied this questionnaire among 114 participants from the communities of Timiskaming and Peel.

**Results:**

We found that tobacco smoking, alcohol consumption, residential use of organochlorine pesticides, and potential exposure to toxic metals were dominant factors among Timiskaming respondents. We found significant differences regarding active smoking, chronic alcohol use, reported indoor and outdoor household pesticide use, and gold and silver mining in the Timiskaming region.

**Conclusions:**

This study, the first to assess environmental factors in the Timiskaming community, identified higher reported exposures to tobacco, alcohol, pesticides, and mining in Timiskaming when compared with Peel. These significant findings highlight the need for specific public health assessments and interventions regarding community environmental exposures.

## 1. Introduction

Colorectal cancer rates follow an unequal population distribution and burden around the world (Henry, Niu, & Boscoe, 2009). The worldwide geographical differences are so great that colorectal cancer incidence rates range by 20 fold, with the lowest incidence in India and the highest incidence in Japan (Adami, Hunter, & Trichopoulos, 2008). Colorectal cancer is the third most diagnosed cancer in males and second in females worldwide, similar to that in Canada where it is the third most common cancer diagnosis and the second leading cause of cancer related deaths (Adami et al., 2008; Canadian Cancer Society, 2011; Centres for Disease Control and Prevention [CDC], 2011). In Ontario, colorectal cancer incidence rates vary prominently from the highest incidence in the North East region of Timiskaming in 2003 (70.4 cases per 100, 000) to the lowest incidence in the Southern region of Peel in 2003 (41.3 cases per 100, 000) (Public Health Agency of Canada [PHAC], 2011). To date, no studies have been conducted in Timiskaming pertaining to cancer incidence. Identification of the factors that contribute to colorectal cancer disparities will help to facilitate preventive interventions, making it possible to reduce its magnitude (Jemal et al.,2011).

Risk factors that are often related to the risk for colorectal cancer incidence are generally classified as non-modifiable or modifiable factors. Non-modifiable factors that are often related to colorectal cancer incidence rates are age, gender, family history, and genetic predisposition (Canadian Cancer Society, 2011). The risk tends to be higher in males than females and increases after the age of 40 years and more so after the age of 50 years (Haggar & Boushey, 2009; Robb, Miles, & Wardle, 2004). With regards to family history and genetic predisposition, only about 20% of cases are recognized to be associated to family history and 5-10% associated to genetic risk (Haggar & Boushey, 2009). Modifiable risk factors that are often discussed with colorectal cancer are diet and body weight. There are evident inconsistencies concerning the relationship between diet and colorectal cancer (Alexander, Cushing, Lowe, Sceurman, & Roberts, 2009; Cho et al., 2004; Park et al., 2005; Sanjoaquin, Allen, Couto, Roddam, & Key, 2004). When examining body weight, obesity has been found to have a strong link to colorectal cancer (Moghaddam, Woodward, & Huxley, 2007). These studies ultimately reveal gaps in the research concerning the remaining portion of colorectal cancer incidence rates. Colorectal cancer may also be linked to modifiable risk factors that are classified as environmental. These environmental risk factors are not nutritional and can be altered or changed as they relate to personal behaviours, lifestyles and occupations. Environmental factors are a missing component of the present knowledge as there is a lack of evidence in the current available literature.

To explore environmental risk factors that may be linked to the high incidence colorectal cancer rate among Timiskaming inhabitants, we first performed a systematic review of non-nutritional modifiable environmental risk factors associated with colorectal cancer incidence. We then developed a questionnaire assessment tool of the factors identified through the systematic review and applied the tool to the community of Timiskaming and the reference community of Peel.

## 2. Methods

### 2.1 Environmental Risk Factor Categories

A systematic review of the published literature regarding non-nutritional modifiable environmental risk factors and colorectal cancer was completed in collaboration with an information specialist (K.M). Our search strategy selected original studies in humans that addressed environmental risk factors with colorectal cancer as the measurable outcome. A comprehensive search of the National Library of Medicine (PubMed) database up until April 12, 2011 was performed using the key words “colorectal neoplasms”, “ethanol”, “alcoholism”, “alcoholic beverages”, “alcoholic drinking”, “smoking”, “tobacco”, “air pollution”, “adverse effects ionizing radiation”, “metals”, “heavy/adverse effects”, “light/adverse effects”, “occupational exposure”, “pesticides” and “organochlorine products”. The initial search yielded 534 citations which were reviewed by the primary investigator utilizing a created inclusion criteria tool. The articles included were then transferred to a data extraction spreadsheet where they were further categorized based on risk factor. These categories were tobacco smoking, alcohol, pesticides, metal toxins and occupational exposures as risk factors for colorectal cancer.

### 2.2 Development and Application of the Environmental Assessment Questionnaire

Following the systematic review, the development of a questionnaire tool was necessary to assess the identified environmental risk factors in the communities of interest. The questionnaire was created using five other questionnaire tools which were standardized, published, and previously utilized with communities in Canada, United States, and Singapore. These questionnaires encompassed different aspects of environmental risk factors assessing community and population exposures. The tools used for our study were the National Health and Nutritional Examination Study (NHANES) (2009-10), Joint Canada/US Survey of Health (JCUSH) (2004), Cape Cod Breast Cancer Study (1999), Canadian Community Health Study (CCHS) (2010), and the Genes and Environment in Lung Cancer Study (2005) (CDC, 2010; Statistics Canada, 2004; Silent Spring Institute, 1999; Statistics Canada, 2010; Lam, 2005). The selected questions were inserted into the new questionnaire and focused on general health, housing and socio-demographics, as well as exposures to tobacco smoke, alcohol, toxic metals, and chemicals in residential and occupational settings. The questionnaire was reviewed by all authors and piloted among 11 individuals with the purpose of determining if the tool was consistent, comprehensible, and time efficient.

For this survey-based ecological approach, individuals 18 years of age and older were invited to participate from the Timiskaming Ontario Early Years Centre and the Mississauga Centre Ontario Early Years Centre. The target population were parents as they are the most frequent visitors of these centres and are recognized as the key providers of health for his or her family. The study was approved by the Research Ethics Board at the University of Ontario Institute of Technology, Oshawa, Ontario. The questionnaire tool was administered to a group of participants and an online printable version and pick up/take home method was also provided for participants. A feedback letter was provided to each participant to inform them of when the study results would be presented to the community after the completion of the project. The Timiskaming location provided 53 completed questionnaires which were all included in the study. The reference location of Peel completed a total of 65 questionnaires, four of which were not applicable due to incompletion or not being returned, totalling to 61 completed questionnaires. Questionnaires deemed as incomplete were those with two or more sections incomplete and thus were excluded from the study. The data from the questionnaires was tabulated and organized by sections. The data was verified by the primary investigator by randomly selecting 4 to 8 questions per section of each questionnaire and confirming if the correct response was transferred from the questionnaire to the spreadsheet.

### 2.3 Data Analysis

The statistical analysis tool SPSS (Statistics Version 19) was utilized for analysis by two authors (J.S & R.K). The data was first normalized to ensure all values were standardized across both community data sets and the data was then coded to simplify the datasets. The coding scheme used to aggregate the survey results were then standardized using binary values for Yes/No answers and the questions containing Likert scales were coded numerically ranging from 1=strongly disagree to 5=strongly agree. For questions containing ‘not applicable’ and ‘do not know’ responses, codes -1 and -2 were used respectively. Normalizing and coding the data sets prepared the data for manipulation. The data was then checked by random data verification to ensure the accuracy of the data entry for all datasets. The corresponding sections from each community group were examined to ensure comparability among sections. Each section was assessed using descriptive statistics and the frequencies were plotted as histograms to identify normalization. Responses that were ‘do not know’ or deemed as a ‘missing value’ were not included in most response values within each category. However, there were cases where the missing values were deemed as ‘not applicable’ these responses however, were usable as a response set.

To determine the differences pertaining to parametric curves, we employed parametric tests such as the independent sample t-test and one way ANOVA. The t-test was used to examine categorical variables with only up to two categories within the questions, values of p < 0.05 were determined to be statistically significant. The one way ANOVA was used when examining categorical or continuous variables with three or more categories and was used for all questions that fit this criterion (Hill & Lewicki, 2007). Normality was observed using the Kolmogorov-Smirnov (KS) non-parametric test. This would ensure that the correct tests had been utilized for the categorical and continuous data. The non-parametric Mann-Whitney U test was the test of choice to evaluate the distribution of the variables with non-uniform normality, as it is a reliable and widely used test.

## 3. Results

A total of 114 participants took part in the study, a majority of them being females between the ages of 25 to 45 years from Timiskaming and Peel. Table 1 demonstrates the socio-demographic findings of the participants. Timiskaming participants were found to report a lower health status (p<0.01) and an overall lower level of education (p<0.001) than Peel participants. Birth place and ethnic background also significantly varied between the communities (p<0.001) as Timiskaming participants demonstrated little diversity and Peel demonstrated significantly more diversity. There were also more participants in Timiskaming with an Aboriginal background when compared to Peel participants (p<0.01). No statistical significant differences were observed regarding age, gender, relationship status, language preference, and total household income.

**Table 1:** Socio-demographic and health characteristics of participant populations (Total sample = 114)

	Timiskaming	Peel	P value
Age			0.972
18-24	4 (7.5%)	2 (3.3%)	
25-45	38 (71.7%)	49 (80.3%)	
46-59	10 (18.9%)	9 (14.8%)	
60-75	1 (1.9%)	1 (1.6%)	
76+	0 (0%)	0 (0%)	
Health Status			0.002
Excellent	4 (7.5%)	10 (16.4%)	
Very Good	16 (30.2%)	29 (47.5%)	
Good	24 (45.3%)	19 (31.1%)	
Fair	7 (13.2%)	3 (4.9%)	
Poor	2 (3.8%)	0 (0%)	
Gender			0.798
Female	47 (88.7%)	55 (90.2%)	
Male	6 (11.3%)	6 (9.8%)	
Relationship			0.623
Married	31 (58.5%)	44 (72.1%)	
Living Common Law	12 (22.6%)	2 (3.3%)	
Living with a partner	2 (3.7%)	1 (1.6%)	
Widowed	1 (1.9%)	2 (3.3%)	
Separated	1 (1.9%)	2 (3.3%)	
Divorced	3 (5.7%)	2 (3.3%)	
Single	3 (5.7%)	8 (13.1%)	
Education			<0.001
Less than High School	2 (3.8%)	0 (0%)	
High School	8 (15.1%)	4 (6.6%)	
Trades certificate/diploma	4 (7.5%)	3 (4.9%)	
Non-university/college certificate	16 (30.2%)	11 (18.0%)	
University or college certificate	11 (20.8%)	9 (14.8%)	
Bachelor degree	9 (17.0%)	21 (34.4%)	
Professional School degree	3 (5.7%)	13 (21.3%)	
Birthplace			<0.001
Asia	2 (3.8%)	22 (36.7%)	
Europe	2 (3.8%)	10 (16.7%)	
Middle East	0 (0%)	3 (5.0%)	
North America	49 (92.5%)	23 (38.3%)	
South America	0 (0%)	2 (3.3%)	
Aboriginal Ethnic Background			0.007
Yes	6 (11.3%)	0 (0%)	
No	47 (88.7%)	61 (100%)	
Ethnic Background			<0.001
Caucasian	51 (96.2%)	21 (34.4%)	
Hispanic or Latino	0 (0%)	2 (3.3%)	
Black or African American	0 (0%)	5 (8.2%)	
South Asian	2 (3.8%)	16 (26.2%)	
East Asian	0 (0%)	14 (23.0%)	
West Asian or Middle Eastern	0 (0%)	2 (3.3%)	
More than one	0 (0%)	1 (1.6%)	
Language			0.153
English	40 (75.5%)	39 (63.9%)	
French	6 (11.3%)	1 (1.6%)	
Other	1 (1.9%)	17 (27.9%)	
More than one	6 (11.3%)	4 (6.6%)	
Total Household Income			0.122
< 25,000	6 (12.2%)	1 (1.8%)	
25,000 < 50,000	14 (28.6%)	9 (16.1%)	
50,000 < 80,000	9 (18.4%)	17 (30.4%)	
80,000 < 100,000	7 (14.3%)	18 (32.1%)	
100,000 +	13 (26.5%)	11 (19.6%)	

### 3.1 Tobacco Smoking Patterns

Table 2 demonstrates the reported active and passive smoking exposures of participants. Significant differences between the communities were observed when participants were asked if they had smoked a whole cigarette in his or her lifetime (p<0.001), age of first whole cigarette smoked (p<0.01), highest number of cigarettes smoked daily (p<0.05), and if others smoked every day or almost every day in the household (p<0.05). No statistically significant differences were found for age of initiation of smoking daily, cessation of smoking, and for any second-hand smoke exposure in the household, public, or vehicle environments.

**Table 2:** Active and passive tobacco smoking patterns (Total sample = 114)

	Timiskaming	Peel	P-value
Smoked a whole cigarette in lifetime			<0.001
No	18 (34%)	42 (68.9%)	
Yes	35 (66%)	19 (31.1%)	
Age of first whole cigarette smoked			0.003
10-13	7 (33.3%)	0 (0%)	
14-17	11 (52.4%)	9 (56.2%)	
18-21	3 (14.3%)	5 (31.2%)	
22-25	0 (0%)	1 (6.3%)	
26-29	0 (0%)	1 (6.3%)	
Current smoking pattern			0.010
Every day	8 (23.5%)	3 (50.0%)	
Some days	1 (3.0%)	3 (50.0%)	
Not at all	25 (73.5%)	0 (0%)	
Age of initiation of smoking daily			0.458
10-13	2 (11.8%)	0 (0%)	
14-17	8 (47.0%)	4 (44.4%)	
18-21	5 (29.4%)	4 (44.4%)	
22-25	2 (11.8%)	1 (11.1%)	
Cessation of daily smoking			0.352
Never smoked every day	1 (6.25%)	0 (0%)	
<1 year ago	1 (6.25%)	0 (0%)	
1 year to < 2 years ago	1 (6.25%)	0 (0%)	
2 years to < 3 years ago	0 (0%)	1 (12.5%)	
3 or more years ago	13 (81.3%)	7 (87.5%)	
Complete cessation of smoking			0.630
< 1 year ago	1 (6.25%)	0 (0%)	
1 year to < 2 years ago	1 (6.25%)	0 (0%)	
2 years to < 3 years ago	0 (0%)	1 (14.3%)	
3 or more years ago	14 (87.5%)	6 (85.7%)	
Others smoking every day or almost every day in the household			0.011
No	3 (25.0%)	11 (73.3%)	
Yes	9 (75.0%)	4 (26.7%)	
Exposure to second hand smoke every day or almost every day in car or vehicle in the past month			0.552
No	46 (88.5%)	56 (91.8%)	
Yes	6 (11.5%)	5 (8.2%)	
Exposure to second hand smoke every day or almost every day in public places in the past month			0.763
No	45 (84.9%)	53 (86.9%)	
Yes	8 (15.1%)	8 (13.1%)	

### 3.2 Alcohol Drinking

Alcohol consumption was assessed and is presented in Table 3. Significant differences between Timiskaming and Peel were identified regarding the number of days during lifetime with at least one drink of alcohol (p<0.05), the number of drinks in the past 12 months (p<0.01), having five or more drinks of alcohol in one occasion in the past 12 months (p<0.001), and for having five or more drinks in a row or in two hours in the past month (p<0.05). No statistically significant differences were present for the age of first drink of alcohol, having a drink of alcohol in the past 12 months, and for having a drink of alcohol in the past week.

**Table 3:** Alcohol drinking patterns (Total sample = 114)

		Timiskaming	Peel	P-value
Age at first drink of alcohol				0.340
	Never had a drink	2 (3.9%)	15 (25.9%)	
	8 years or younger	1 (2.0%)	0 (0%)	
	9 or 10 years old	1 (2.0%)	0 (0%)	
	11 or 12 years old	4 (7.8%)	0 (0%)	
	13 or 14 years old	17 (33.3%)	4 (6.9%)	
	15 or 16 years old	12 (23.5%)	14 (24.1%)	
	17 years old or older	14 (27.5%)	25 (43.1%)	
Number of days during lifetime with at least one drink of alcohol				0.015
	1 or 2 days	2 (4.6%)	2 (5.0%)	
	3 to 9 days	4 (9.3%)	3 (7.5%)	
	10 to 19 days	0 (0%)	7 (17.5%)	
	20 to 39 days	4 (9.3%)	9 (22.5%)	
	40 to 99 days	6 (14.0%)	9 (22.5%)	
	100 or more days	27 (62.8%)	10 (25.0%)	
Alcohol beverages in the past 12 months				0.079
	No	8 (15.7%)	14 (31.1%)	
	Yes	43 (84.3%)	31 (68.9%)	
Number of drinks in the past 12 months				0.004
	Less than once a month	15 (35.7%)	18 (58.1%)	
	Once a month	3 (7.1%)	6 (19.3%)	
	2 or 3 times a month	9 (21.4%)	4 (12.9%)	
	Once a week	4 (9.5%)	1 (3.2%)	
	2 to 3 times a week	10 (23.8%)	2 (6.5%)	
	4 to 6 times a week	1 (2.4%)	0 (0%)	
	Every day	0 (0%)	0 (0%)	
5 or more drinks of alcohol in one occasion in the past 12 months				<0.001
	Never	17 (40.5%)	24 (75.0%)	
	Less than once a month	13 (31.0%)	8 (25.0%)	
	Once a month	4 (9.5%)	0 (0%)	
	2 to 3 times a month	4 (9.5%)	0 (0%)	
	Once a week	3 (7.1%)	0 (0%)	
	More than once a week	1 (2.4%)	0 (0%)	
Number of days in the past month with a drink of alcohol				0.090
	None	13 (31.0%)	8 (25.8%)	
	1 or 2 days	10 (23.8%)	16 (51.6%)	
	3 to 5 days	9 (21.4%)	5 (16.1%)	
	6 to 9 days	5 (11.9%)	2 (6.5%)	
	10 to 19 days	4 (9.5%)	0 (0%)	
	20 to 29 days	1(2.4%)	0 (0%)	
	All 30 days	0 (0%)	0 (0%)	
5 or more drinks in a row or in two hours in the past month				0.032
	None	32 (74.4%)	28 (87.5%)	
	1 day	3 (7.0%)	4 (12.5%)	
	2 days	2 (4.7%)	0 (0%)	
	3 to 5 days	5 (11.6%)	0 (0%)	
	6 to 9 days	0 (0%)	0 (0%)	
	10 to 19 days	0 (0%)	0 (0%)	
	20 or more days	1 (2.3%)	0 (0%)	
Alcohol beverages in the past week				0.257
	No	24 (55.8%)	22 (68.7%)	
	Yes	19 (44.2%)	10 (31.3%)	

### 3.3 Household Pesticide Use

Table 4 presents significant differences between the communities regarding their reported use of insecticides at the home/residence (p<0.01), having a lawn at the current home/residence (p<0.001), the use of chemical products to regularly control mould or mildew (p<0.01), and the use of paint thinner/stripper at the home/residence (p<0.05). There were no statistically significant differences for the time frame and number of times using insecticides, the use of various chemicals on the lawn, the use of professional pesticide lawn services, and the use of pesticides/insecticides/herbicides by the participant or other household members.

**Table 4:** Household pesticide use (Total sample = 114)

		Timiskaming	Peel	P-value
Use of insecticides at current home/residence				0.008
	No	33 (67.3%)	49 (89.1%)	
	Yes	16 (32.7%)	6 (10.9%)	
Number of times insecticides were used at current home/residence				0.772
	Never	0 (0%)	1 (14.3%)	
	Once or twice	11 (78.6%)	4 (57.1%)	
	3 to 10 times	3 (21.4%)	2 (28.6%)	
	More than 10 times	0 (0%)		0 (0%)	
Lawn at current home/residence				<0.001
	No	2 (4.0%)	20 (33.3%)	
	Yes	48 (96.0%)	40 (66.7%)	
Use of chemicals on lawn at current home/residence				0.879
	No	21 (52.5%)	19 (54.3%)	
	Yes	19 (47.5%)	16 (45.7%)	
Total number of times lawn was treated with chemicals				0.354
	Once or twice	17 (89.5%)		13 (81.3%)	
	3 to 20 times	2 (10.5%)		2 (12.5%)	
	More than 20 times	0 (0%)		1 (6.2%)	
First time lawn was treated with chemicals				0.988
	1996-1999	1 (5.3%)	0 (0%)	
	2000-2003	1 (5.3%)	3 (27.3%)	
	2004-2007	7 (36.8%)	1 (9.1%)	
	2008-2011	10 (52.6%)	7 (63.6%)	
Most recent time lawn was treated with chemicals				0.822
	1996-1999	1 (5.3%)	0 (0%)	
	2000-2003	1 (5.3%)	2 (18.2%)	
	2004-2007	5 (26.3%)	1 (9.1%)	
	2008-2011	12 (63.1%)	8 (72.7%)	
Use of chemicals like pesticides/insecticides/herbicides/weed killers household members				0.082
	No	33 (75.0%)		48 (88.9%)	
	Yes	11 (25.0%)	6 (11.1%)	
Use of chemical products regularly to control mould or mildew				0.003
	No	33 (62.3%)	52 (86.7%)	
	Yes	20 (37.7%)	8 (13.3%)	
Use of paint thinner or paint stripper at current home/residence				0.015
	No	33(64.7%)	51 (85.0%)	
	Yes	18 (35.3%)	9 (15.0%)	

### 3.4 Other Factors

We also found differences between Timiskaming and Peel regarding factors concerning metals and occupational exposures. As the Timiskaming region has had silver and gold mining, 52.0% of participants identified gold mining and 23.9% of participants identified silver mining near or within his or her residence. When compared with Peel Region, more Timiskaming participants indicated their household being built prior to 1978 (p<0.001) and reported having had past or current dental amalgam (p<0.001). When asked about occupational exposures, more Timiskaming participants than Peel participants reported working in occupations involving cooking (n=29 vs. n=19), industrial food processing (n=10 vs. n=2), contact with exhaust fumes from vehicles (n=17 vs. n=9), and with burning of coal/wood/oil (n=5 vs. n=0).

## 4. Discussion

This is the first study exploring environmental factors that may be associated with the high incidence of colorectal cancer in Timiskaming, Ontario. Our survey particularly investigated reported exposures to non-nutritional lifestyle, behavioural, occupational and geographical factors, in comparison to a reference population with the lowest incidence of colorectal cancer in Ontario. Our study found that tobacco smoking and alcohol drinking patterns were significantly different between the communities. Other reported differences related to residential use of pesticides and knowledge of mining exposures.

The socio-demographic and health characteristics of Timiskaming and Peel vary in particular areas such as education, birth place, ethnic background, Aboriginal background, and self-reported health status. These were expected differences, excluding the self-reported health status. As health status was a part of the survey administered to participants, it was regarded as their own perception of health. Assessing this provided knowledge that Timiskaming participants varied in how they viewed their own health reporting from ‘excellent’ to ‘poor’, whereas a majority of Peel participants reported from ‘excellent’ to ‘good’. This self-reported health status not only identifies participant perception on health but may also represent each participant’s actual health status.

Regarding tobacco smoking, there were significant differences between the communities concerning age of first whole cigarette smoked, current smoking pattern, the highest number of cigarettes smoked daily, and for other household member smokers smoking every day or almost every day. A comprehensive meta-analysis observed similar findings with an overall increased risk in relation to cigarette smoking and significant associations between variables of daily cigarette smoking, duration, pack years, age of initiation, and colorectal cancer (Liang, Chen, & Giovanni, 2009). Another meta-analysis also found a significant relationship between cigarette smoking and colorectal cancer (Chen, Qiu, Zhang, & Zhao, 2003). Tobacco smoke has been linked to specific forms of cancer, especially lung cancer; however the link to colorectal cancer is less determined. Cigarette smoke may be responsible for the formation and growth rate of adenomatous polyps that can lead to colorectal cancer, particularly with long term smoke exposure (Haggar & Boushey, 2009). The influence of tobacco smoke on cancer can be due to the roughly 4000 carcinogenic chemicals present in the tobacco (Liang et al.,2009).

With alcohol exposure, significant differences between the communities were found for the number of days during lifetime with at least one drink of alcohol, number of drinks in the past 12 months, having five or more drinks of alcohol in one occasion in the past 12 months, and for having five or more drinks in a row or in two hours in the past month. A meta-analysis of five Japanese cohort studies found a clear dose response relationship between alcohol consumption and colorectal cancer risk in males (Mizoue et al., 2008). Furthermore, another meta-analysis found that high alcohol intake was associated with risk of colon cancer when comparing the highest and lowest categories of alcohol intake (Moskal, Norat, Ferrari, & Riboli, 2006).

The use of pesticides and organochlorines demonstrated significant differences regarding insecticide use at the home/residence, having a lawn at the current home/residence, the most recent time of use of professional lawn service using pesticides/insecticides/weed killers, and the use of chemical products to control mould or mildew in the household. The risks with metal toxins presented significant differences when participants were asked about knowledge of silver or gold mining taking place near or within the community, if the home were built prior to 1978, having a dental amalgam, and the time frame of having this dental amalgam. The Timiskaming region was known for silver mining in the 1900s and is now known for its gold mining. Silver mining was commonly extracted with the use of nickel and arsenic compounds and gold mining was extracted using mercury metal. The corresponding mine tailings and waste rock possibly expose the community to metal toxins.

In our study, a majority of participants were females between the ages of 25 to 45 years, indicative of the group of individuals who often used the participant community centres. Self-reported health status among both communities presented a significant difference as Timiskaming participants reported a generally lower health status than those in Peel. This was an unexpected finding that may relate self-perceived health to the actual health of these participants. Education status among participant communities significantly varied, however income level did not. These two factors are often associated with one another to account for socioeconomic status. This may suggest that education and income level are not associated to one another in each of the participant communities.

There is speculation that as communities migrate to host nations, environmental influences may alter the colorectal cancer rates in these migrant communities towards that of the host nation (Kim, Masyn, Kawachi, Laden, & Colditz, 2010). Western Europe, North America, and Australia have shown to have higher incidence of colorectal cancer rates in comparison to South Asia, Latin America, and Africa. As populations of the latter nations migrate to the former nations, the incidence among these migrated communities becomes similar to that of the nations they migrated to (Virk, Gill, Yoshida, Radley, & Salh, 2010). The participants from Timiskaming were primarily of Caucasian background and of North American (Canadian) descent. The Peel participants, on the other hand, were primarily of Caucasian, South Asian, and East Asian background. The high ethnic diversity and large population size of Peel (Statistics Canada, 2011a) may contribute to the low risk of colorectal cancer rates, as the population is constantly changing. Birth place and ethnic background tie together and represent the main differences between the two communities. Timiskaming also has a low Aboriginal participant population which was still significantly different from Peel, as the latter community had no self-identifying Aboriginal participants. This difference may not be significant as it is a very small difference but it does relate to the general population statistics of both communities where only 5.6% of the entire Timiskaming population and 0.5% of the entire Peel population are of Aboriginal descent (Statistics Canada, 2011a; Statistics Canada, 2011b).

Our study presented different strengths, notably as it is the first study performed in Timiskaming, an Ontario region with high incidence of colorectal, breast and lung cancer relative to other provincial regions. This primary study assessed the possible relationship between non-nutritional modifiable environmental risk factors and colorectal cancer at a community level. It provided insight on the potential non-nutritional modifiable environmental risk factors and the further diverging disparities between Timiskaming and Peel. Our study allowed for recognition of potential environmental risk factors in the community of Timiskaming, contributing to enhancing community and individual knowledge, understanding, and empowerment.

There are evident limitations inherent to this survey-based ecological study. Two community centres were utilized as a convenience sample and this may have excluded members of the community who were not in access of the community centres. This relates to the most important limitation of this study which is the small sample size. The Timiskaming community centre had two satellite centres which helped to be more inclusive, however only one centre in Peel was used so this may have prevented those living outside the region of the centre to participate. As a questionnaire tool was used as the primary method of data collection, this may have presented recall bias as participants may not have been able to remember exposures or they may have answered questions inaccurately. The questionnaire tool was only printed in English, creating a limitation for other language users. A limitation in the recruitment process through the use of community centres is that a majority of the participants from the centres were females excluding male populations.

The findings from this study indicate that there are dominant non nutritional modifiable environmental risk factors in the region of Timiskaming, specifically regarding tobacco smoking, alcohol use, household chemical use, and mining exposures. These may contribute to the high colorectal cancer incidence rates and colorectal cancer disparities present in Ontario communities. Our findings are important for future research as disparities in cancer incidence are present not only in Ontario but in many regions of Canada. Our study contributes to knowledge on communities like Timiskaming where cancer risks are higher than in other communities and where environmental risks are less acknowledged. Future research can stem from our study to incorporate a more rigorous approach and utilize better methodology. It is recommended that future studies utilize cohort study designs accompanied by collaborating with community health units, looking at disparities across provincial boundaries, expanding data collection methods, and addressing other factors affecting incidence such as screening programs or barriers to accessing health services. Ultimately, community cancer disparities may be reduced by understanding and examining the contributing environmental risk factors.
